# Application of Metabolic Engineering to the Production of Scopolamine

**DOI:** 10.3390/molecules13081722

**Published:** 2008-08-18

**Authors:** Javier Palazón, Arturo Navarro-Ocaña, Liliana Hernandez-Vazquez, Mohammad Hossein Mirjalili

**Affiliations:** 1 Laboratori de Fisiologia Vegetal, Facultat de Farmacia, Universitat de Barcelona, Avda Diagonal 643, E-08028 Barcelona, Spain; E-mail: lilianahernandezv@ub.edu; 2 Departamento de Alimentos y Biotecnologia, Facultad de Química, Universidad Nacional Autónoma de México, México DF, México; E-mail: arturono@servidor.unam.mx; 3 Medicinal Plants and Drugs Research Institute, Shahid Beheshti University, Evin, Tehran, Iran; E-mail: m-mirjalili@sbu.ac.ir

**Keywords:** Scopolamine, hyoscyamine, hairy roots, putrescine *N*-methyltransferase, hyoscyamine-6-β-hydroxylase

## Abstract

Scopolamine is an alkaloid widely used in medicine for its anticholinergic activity. The aim of this review is to show that metabolic engineering techniques constitute a suitable tool to improve the production of tropane alkaloids, focusing in particular on scopolamine. We present an overview of results obtained by various research groups, including our own, who have studied the overexpression of genes involved in the biosynthesis of scopolamine in different plant species that produce tropane alkaloids. Experiments carried out to improve production in hairy root cultures will also be described, as well as those attempting to biotransform hyoscyamine into scopolamine in roots and transgenic tobacco cells.

## Introduction

In developing countries, plants are the main source of medicines: according to the World Health Organization, as much as 80% of the world’s population in developing countries relies on traditional medicine for its primary health care, and this is mainly based on plant remedies. The use of herbal medicine in developed countries is also growing; for example, it has been reported that 25% of the UK population regularly takes herbal medicines [1]. Bioactive compounds currently extracted from plants, besides being used as important therapeutic and remedy products, also serve as food additives, pigments, dyes, insecticides, cosmetics and perfumes. However, the fast rate at which the habitats for medicinal plants are disappearing, together with geopolitical problems, has resulted in certain plant-derived compounds becoming increasingly difficult to obtain. 

Advances in biotechnology, particularly methods for culturing plant cells and organs, and for micropropagation of medicinal plants will provide new means for the commercial processing of even rare plants and the chemicals they provide. Metabolic engineering involves the targeted and purposeful alteration of metabolic pathways found in an organism to achieve better understanding and use of cellular pathways for chemical transformation, energy transduction, and supramolecular assembly [2]. This technique applied to plants will permit endogenous biochemical pathways to be manipulated, resulting in the generation of transgenic crops in which the range, scope, or nature of a plant’s existing natural products are modified to provide beneficial commercial, agronomic and/or post-harvest processing characteristics [3].

Over the last decades, plant cell cultures have been intensively investigated as a possible tool for the production of commercial plant secondary metabolites, including fine chemicals such as pharmaceuticals, agrochemicals, flavors, insecticides, fragrances and cosmetics [4]. In spite of the efforts in the field of *in vitro* production of phytochemicals, few industrial processes have been developed, involving only a limited number of secondary products, such as shikonin, berberine, ginsenosides and paclitaxel [5]. As in many cases production is too low for commercialization, metabolic engineering can provide various strategies to improve productivity, such as:

- Increasing the number of producing cells
- Increasing the carbon flux through a biosynthetic pathway by overexpression of genes codifying for rate-limiting enzymes or blocking the mechanism of feedback inhibition and competitive pathways
- Decreasing catabolism

**Figure 1:**
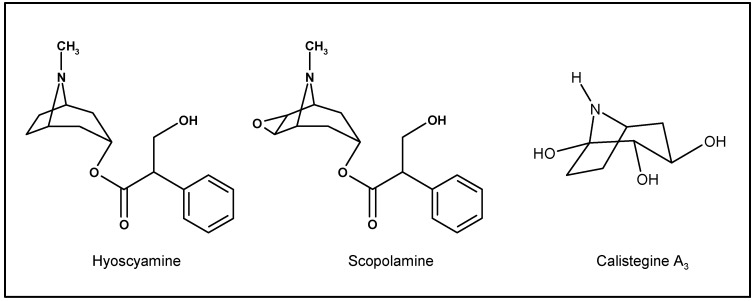
Chemical structures of the anticholinergic alkaloids hyoscyamine, scopolamine and calystegine A3.

Many of the isolated pure compounds with biological activity are alkaloids, a diverse group of nitrogen-containing chemical ring structure compounds, with alkali-like chemical reactivity and pharmacological activity. Although the pharmacological effects of alkaloids have been studied, the biosynthetic pathways of these compounds are still obscure. Among the most famous are the tropane alkaloids, such as (-)-hyoscyamine, its racemate atropine, and scopolamine (hyoscine), which have an 8-azabicyclo[3.2.1]octane esterified nucleus (Figure 1). These alkaloids are commonly found in plants of different families: Solanaceae, Erythroxylaceae, Convolvulaceae, Proteaceae, Euphorbiaceae, Rhizophoraceae and Cruciferae [6]. Related to the tropane alkaloids, a new group of nortropane alkaloids, the calystegines, was discovered only 15 years ago. Calystegines bear three to five hydroxyl groups in various positions, making them water-soluble (Figure 1), and they share metabolic steps and enzymes of the formation of tropane alkaloids.

The tropane alkaloids, scopolamine and its precursor hyoscyamine, which are found mainly in the *Duboisia*, *Datura*, *Hyoscyamus,* *Atropa* and *Scopolia* species (see Table 1), together with their semisynthetic derivatives, are used as parasympatholytics that competitively antagonize acetylcholine. Anticholinergics are generally used as mydriatics, and to control the secretion of saliva and gastric acidity, slow gut motility and prevent vomiting. The roots are the principal site of alkaloid biosynthesis but leaves can also accumulate significant quantities of these compounds. Hyoscyamine is normally the more abundant alkaloid in plants, with scopolamine produced in greater quantities only in Duboisia spp and *Datura metel* [7]. The main source of raw material for tropane alkaloid production worldwide is *Duboisia* leaves, which contain 2-4% of total alkaloids, with more than 60% of scopolamine and 30% of hyoscyamine. Conventional cultivation of some varieties that can accumulate up to 6% of scopolamine has been established in Australia, Ecuador and Brazil, producing 1 t/ha of this alkaloid.

Scopolamine is the most valuable tropane alkaloid, preferred for its higher physiological activity and fewer side effects. The world demand for this alkaloid is estimated to be about 10 times greater than for hyoscyamine and its racemic form atropine. Accordingly, there has been a long-standing interest in boosting the scopolamine content of producing plants and their *in vitro* cultures. In the case of biotechnological production of scopolamine, increasing the number of cells able to produce this compound is essential because tropane alkaloids are mostly synthesized in young cells and translocated to the aerial parts of the plants [8], so biotechnological processes based on undifferentiated systems such as calli or cell cultures are not productive [9,10]. Thus the attempts to produce scopolamine in biotechnological systems are based mainly on hairy root cultures.

## Biosynthesis of scopolamine

**Figure 2:**
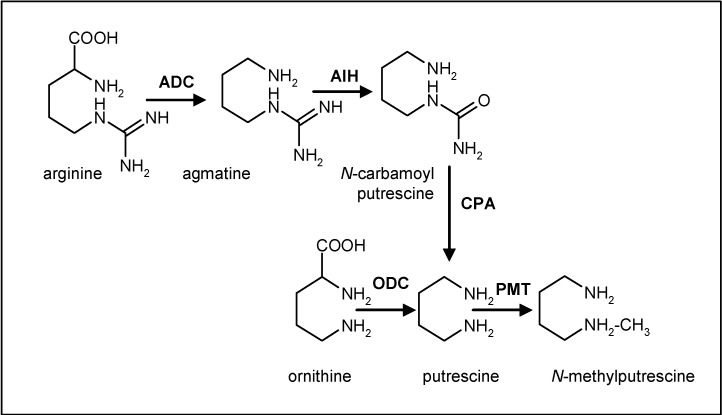
Biosynthesis of *N*-methylputrescine from arginine and ornithine. ADC: Arginine decarboxylase. AIH: Agmatine iminohydrolase. CPA: N-carmaboylputrescine amidohydrolase. ODC: Ornithine decarboxylase. PMT: putrescine N-methyltransferase.

At present, the metabolic pathway for scopolamine biosynthesis is still not fully understood and only a few of the enzymes involved have been isolated and corresponding genes cloned, while practically nothing is known about how the synthesis is regulated [11]. The tropane ring present in the molecule of scopolamine derives from putrescine via an *N*-methylpyrrolinium salt. Walton *et al.* [12] demonstrated that two amino acids, ornithine and arginine, are involved in the biosynthesis of putrescine by alternative pathways (Figure 2). The decarboxylation of ornithine yields putrescine directly, whereas arginine has to be transformed into agmatine to produce putrescine (see Figure 2). 

**Table 1:** Distribution of hyoscyamine and/or scopolamine in the Solanaceae family [6].

Subfamily	Tribe	Genus	Species
Solanoidae	Daturae	*Datura*	*D. stramonium, D. ferox, D. quercifolia, D. pruinosa, D. leichahhardtii, D. inoxia, D. discolor, D. metel, D. wrightii*
		*Brugmansia*	*B. aurea, B. sanguinea, B. arborea, B. xcandida, B. xdolichocarpa, B. xinsignis, B. versicolor, B. vulcanicola*
	Solandrae	*Solandra*	*S. longifolia, S. grandifolia, S. guttata, S. hartvegii, S. hirsute, S. macranthe*
	Solaneae	*Atropa*	*A. belladonna*
		*Latua*	*L. pubiflora*
		*Acristus*	*A. arborea*
		*Mandragora*	*M. autumnale, M. vernalis*
		*Salpichroa*	*S. organiflora*
	Hyoscyameae	*Scopolia*	*S. carniolica, S. parviflora*
		*Hyoscyamus*	*H. muticus, H. niger, H. albus, H. aureus*
		*Physochlaina*	*P. physaloides, P. orientalis*
		*Przewalskia*	*P. tangutica*
Cestroideae	Anthocercidae	*Duboisia*	*D. hopwoodii, D. leichhardtii, D. myoporoides, D. arenitensis, D. hybrid*
		*Anthotroche*	*A. myoporoides, A. pannosa, A. walcottii*
		*Anthocercis*	*A. littorea, A. viscose, A. fasciculote, A. ilicitolia, A. genistoides*
		*Cyphanthera*	*C. anthocercidea, C. albicans*
		*Symonanthus*	*S. aromaticus*
		*Grammosolen*	*G. dixonii*
		*Crenidium*	*C. spinescens*

**Figure 3:**
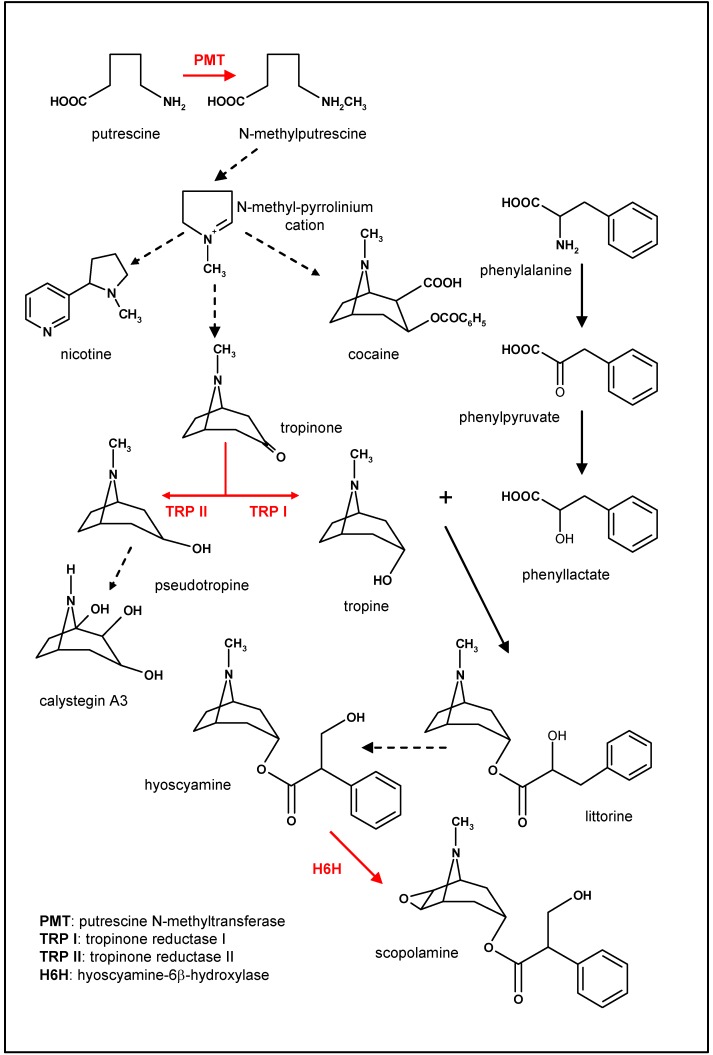
Overview of the most important steps in the scopolamine biosynthetic pathway. The enzymes overexpressed in scopolamine-producing hairy root cultures are in red.

The next step is the *S*-adenosyl-methionine (SAM)-dependent methylation of putrescine catalysed by putrescine *N*-methyltransferase (PMT; EC2.1.1.53), forming *N*-methylputrescine. This enzyme removes putrescine from the polyamine pool and drives the methylated compound exclusively toward alkaloid production (Figure 3). Feeding experiments [13,14,15] have demonstrated that this is a flux limiting step. The oxidative transamination of *N*-methylputrescine yields an aminoaldehyde that is primed for cyclisation to an *N*-methylpyrrolinium intermediate [16].

The next steps in the biosynthesis require the condensation of an appropriate acetate-derived intermediate with *N*-methylpyrrolinium. Hygrine, an alkaloid that is formally the product of an acetone condensation with *N*-methylpyrrolidine, was for many years considered to be an intermediate in the biosynthesis of scopolamine, but recent studies suggest that hygrine is not involved in tropane ring biosynthesis [16]. Two tropinone reductases responsible for tropane alcohol formation have been isolated and characterized from root cultures of several species (see [17] and references therein). All the plant species studied so far possess two strictly stereospecific tropinone reductase activities, one (TRI) producing tropine and the other (TRII) producing pseudotropine (see Figure 3). Pseudotropine, as a product of tropinone reduction, undergoes a rapid conversion to calystegines, while tropine is esterified to yield hyoscyamine, the precursor of scopolamine [19]. 

The biosynthesis of tropic acid, the ester moiety of scopolamine, has been a topic of interest for many years [19,20,21,17]. Isotopic labelling studies in transformed root cultures of *Datura stramonium* have demonstrated that littorine [(*R*)-phenyllactoyl tropine] is the direct biosynthetic precursor of hyoscyamine by an intramolecular rearrangement [22] (Figure 3). Thus, the tropate ester moiety of hyoscyamine is derived by isomerisation from the (*R*)-phenyllactate ester of littorine. However, the results obtained by Patterson and O’Hagan [23] suggest that the rearrangement of littorine into hyoscyamine does not occur with a vicinal interchange process as previously thought, and thus the mechanism of isomerisation remains to be determined. Recently, a multifunctional cytochrome P450 capable of littorine rearrangement via a hyoscyamine aldehyde has been identified from *Hyoscyamus niger* [24].

Scopolamine, the 6,7-epoxide of hyoscyamine, is formed by direct oxidation of hyoscyamine without the intermediacy of a double bond. Hyoscyamine 6β-hydroxylase (H6H; EC 1.14.11.11) is an oxoglutarate-dependent dioxygenase that mediates a two-step reaction to generate the epoxide. The first step involves a hydroxylation to lead 6β-hydroxyhyoscyamine and the same enzyme mediates the epoxide ring closure to generate scopolamine [17].

## Hairy root cultures as a source of scopolamine

The hairy root system based on inoculation with *Agrobacterium rhizogenes* has become popular in the two last decades as a method of producing secondary metabolites synthesized in plant roots [25,26]. Unorganized plant tissue cultures are frequently unable to produce secondary metabolites at the same levels as the intact plant. This is also the case of scopolamine production in undifferentiated *in vitro* cultures of Solanaceae, probably due to the specific location of some of the key enzymes involved in this biosynthetic pathway [27]. Suzuki *et al.* [28] have demonstrated that the expression of the *pmt* gene was pericycle-specific, and it has also been shown that H6H is localized in the root pericycle [29,30]. In addition, Nakajima and Hashimoto [31] have observed that TR proteins accumulate in the lateral roots of *Hyoscyamus niger*. Another possible reason for the low production of scopolamine in undifferentiated *in vitro* cultures could be that the auxin added to the callus and cell culture media for normal growth inhibits the activity of some of the key enzymes involved in scopolamine biosynthesis, such as PMT [32].

The hairy root phenotype is characterized by fast hormone-independent growth, lack of geotropism, lateral branching and genetic stability. The secondary metabolites produced by hairy roots arising from the infection of plant material by *A. rhizogenes* are the same as those usually synthesized in intact parent roots, with similar or higher yields [33]. This feature, together with genetic stability and generally rapid growth in simple media lacking phytohormones, makes them especially suitable for biochemical studies not easily undertaken with root cultures of an intact plant. 

During the infection process *A. rhizogenes* transfers a part of the DNA (transferred DNA, T-DNA) located in the root-inducing plasmid Ri to plant cells and the genes contained in this segment are expressed in the same way as the endogenous genes of the plant cells [34]. Some *A. rhizogenes*, such as strain A4, have the T-DNA divided in two sections, the TR-DNA and TL-DNA, each of which can be incorporated separately into the plant genome. Two sets of pRi genes are involved in the root induction process: the *aux* genes located in the TR region of the pRi T-DNA and the *rol* (root loci) genes of the TL region [35]. The *ags* genes responsible for opine biosynthesis in the transformed tissues are also located in the TR region [36]. Opines are synthesized by plant transformed cells and are only used by *Agrobacterium* as a source of nitrogen and carbon.

Due to the similarities of the *A. rhizogenes* and *A. tumefaciens* infection processes, and because both microorganisms are very closely related, it has been suggested that the most important *A. rhizogenes* oncogenes encode proteins involved in the regulation of plant hormone metabolism. *Aux* genes provide transformed cells with an additional source of auxin [37,38], but they do not seem essential for developing hairy root disease [39]. However, *rol* genes have functions that are most likely other than that of producing mere alterations in plant hormone concentrations [40].

Several authors have investigated the effect of TR and TL regions of *A. rhizogenes* on growth and morphology of transformed roots and plants, but until now there have been few studies on the direct effects of oncogenes on secondary metabolism. As has been previously reported, a correlation exists between the expression of the *rol*C gene and tropane alkaloids [41,42,43], *Catharanthus roseus* alkaloids [44], and ginsenoside production [45]. No correlation between *rol*A and *rol*B expression and secondary metabolism was found in any of these studies. Moyano *et al.* [46] showed that the inoculation of leaf sections of tobacco, *Duboisia* hybrid and *Datura metel* plants with the A4 strain of *A. rhizogenes* induced transformed roots with the capacity to produce putrescine-derived alkaloids such as nicotine, hyoscyamine and scopolamine. In general, the obtained hairy roots presented two morphologies: typical hairy roots with a high capacity to produce alkaloids, and callus-like roots with faster growth and lower alkaloid production. The *aux*1 gene of *A. rhizogenes* located in the TR-DNA of *A. rhizogenes* was detected in all roots showing callus-like morphology. However, this gene was only detected in 25-60% of the established root cultures showing typical hairy morphology. These results demonstrate a significant role of *aux* genes in the morphology of transformed roots and the importance of typical hairy root morphology in the production of scopolamine. The studies with *Panax ginseng* hairy roots also support the effects of the genes located in the TR-DNA on root morphology and secondary metabolism [47].

**Figure 4:**
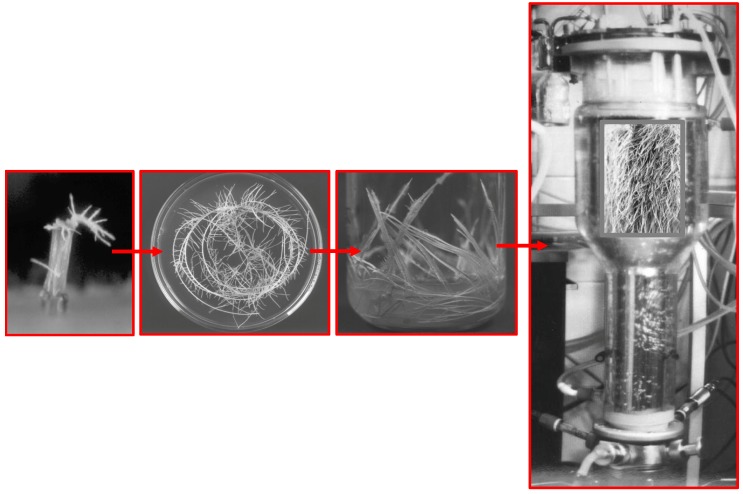
The main steps for the establishment and culture of hairy roots from *Datura metel*.

The hairy roots are normally induced on aseptic, wounded parts of plants by inoculating them with *A. rhizogenes*. In scopolamine-producing Solanaceae plants, roots usually emerge at the inoculation sites after 1-4 weeks (Figure 4), but in the case of other plant species such as *Taxus* it can be more than 4 months before the roots emerge [48]. Root tips are cultured separately in a hormone-free medium, the most commonly used being MS (Murashige-Skoog, [49]), Gamborg’s B5 [50] or SH [51]. The next step for establishing hairy root cultures is to select and characterize the root clones according to their capacity for growth and production of the desired compounds. Sevón *et al*. [33] reported the productivity of scopolamine in more than 15 species, amounts ranging from 0.2 to 32 mg/g DW. Sometimes these productions were achieved after a laborious process to optimize the growth conditions, such as the selection of the more productive clones, and optimization of the production conditions by testing different ionic concentrations as well as the carbon source and pH of the medium.

The treatment with biotic or abiotic elicitors, such as methyl jasmonate, chitosan, salicylic acid or silver nitrate, can also improve the production of tropane alkaloids [11], and references therein, but in some cases the effect of the elicitor is due to an enhancement of cell permeability, which may increase the formation of secondary products by inhibiting operative feedback mechanisms or intracellular degradation of the products [52]. In this context, Cusidó *et al.* [53] have reported that treatment with the permeabilizing agent Tween-20 increases the production of scopolamine in hairy root cultures of *D. metel*. 

The main constraint for the commercial exploitation of hairy root cultures is scaling up to an industrial level, since it has become clear that standard bioreactors are not suitable vessels to achieve this. The uneven distribution of biomass in the vessel does not permit the growth of interconnected tissues, which results in cell necrosis. The growth behavior of the roots also hampers the inoculation, harvesting and sampling procedures. Furthermore, in order to protect the root integrity, the design of mechanical stirred bioreactors should be modified by including stainless or nylon meshes to protect against mechanical shear [54].

**Figure 5:**
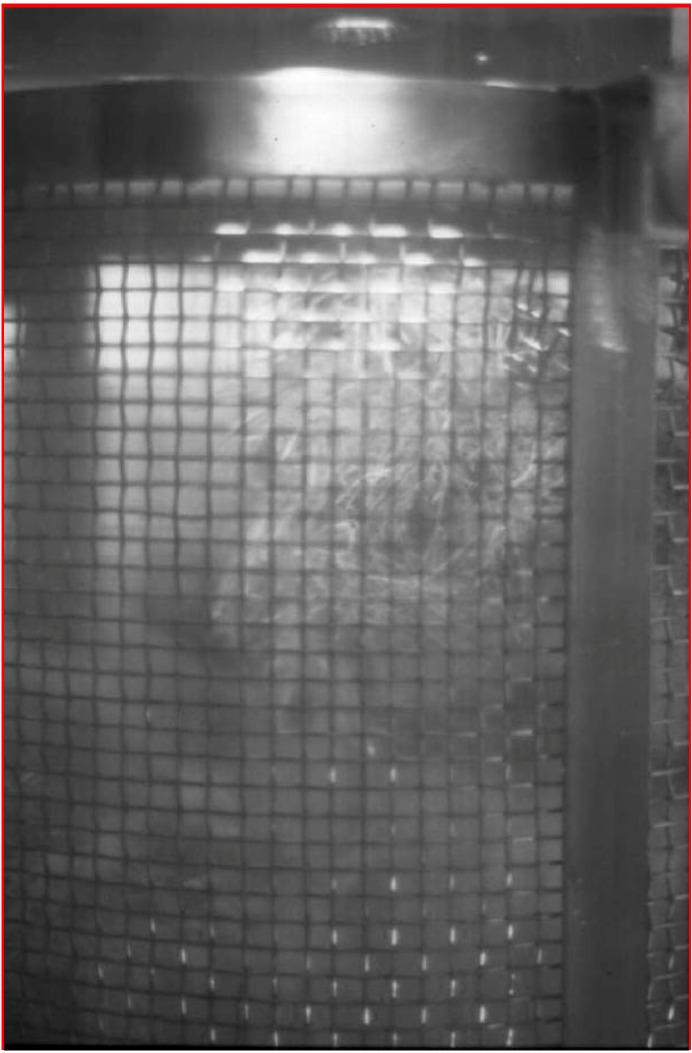
Detail of a hairy root culture of *Duboisia* hybrid showing the anchorage of the roots in the stainless mesh added to protect the culture in the stirred system.

Srivastava *et al.* [55] have recently summarized the attempts to adapt bioreactor design to hairy root cultures; stirred tank, airlift, bubble columns, connective flow, turbine blade, rotating drum, as well as different gas phase reactors have all been used successfully. In the case of tropane alkaloids, different types of bioreactors are used for scopolamine production (Figure 5). Wilson [56] describes the only large droplet bioreactor system with a volume of 500 L designed for hairy root cultures of *Datura stramonium*. On a smaller scale, modified airlift and stirred tanks have been used for scopolamine production in hairy root cultures of *D. metel* [53], connective flow reactors for *H. muticus* [57] and *Atropa belladonna* [58] and more recently a bubble column bioreactor has been employed for root cultures of *Scopolia parviflora* [59]. One such advance is the development of disposable wave bioreactor systems, whose working principle is based on wave-induced agitation, which significantly reduces stress levels. This type of bioreactor has been successfully used for *H. muticus* and *Panax ginseng* hairy root cultures [60].

## Overexpression of the *pmt* gene to improve scopolamine production

It is known that tropane alkaloids are derived from putrescine via *N*-methylputrescine, and that putrescine can also be metabolized into polyamines such as spermidine and spermine. As previously mentioned, the formation of *N*-methylputrescine is catalyzed by putrescine *N*-methyltransferase, which is the first committed step in the biosynthesis of these alkaloids (Figure 2). This suggests that scopolamine production by plant cell cultures can be improved by overexpressing the *pmt* gene. This reaction is common to both tropane and tobacco alkaloids. The cDNA of *pmt* has been cloned from tobacco and *Nicotiana sylvestris* and the enzyme PMT has been purified from tobacco plant roots, where its activity has been measured [61]. Root cultures of *Datura stramonium*, [12,62,63], *Hyoscyamus albus* [64,65] and *H. niger* [66] contain PMT with similar properties to the tobacco enzyme and the expression of the gene has been found in the root pericycle of *Atropa belladonna* [28] as well as in the endodermis, xylem and outer cortex cells of *Nicotiana sylvestris* [67]. In tobacco, PMT is stress-responsive and inducible by methyl jasmonate [67,68,69,70] but, in contrast to the tobacco PMT promoter, in *Atropa belladonna* no jasmonate-responsive element has been identified in the promoter region [28], and correspondingly, hyoscyamine is not enhanced by elicitation in root cultures of *A. belladonna* [18].

The key role of PMT in tropane alkaloid biosynthesis converts this enzyme into a prime target for metabolic engineering, and there have been many attempts to increase the scopolamine production by over-expressing the *pmt* gene. In most cases, the plant material has been transformed with a heterologous gene, such as the *pmt* gene from tobacco, under the control of the CaMV35-S promoter, with the advantages of no feedback inhibition by downstream products, and a higher affinity for the substrate [71]. PMT-overexpressing plants of *A. belladonna* and *N. sylvestris* have already been produced by Sato *et al.* [72]. These authors report an opposite effect of the transgene expression, since no changes were observed in the *Atropa* alkaloid content, while the nicotine content in *N. sylvestris* leaves increased significantly. 

In order to increase the bioproduction of hyoscyamine and scopolamine alkaloids, we have been working with two scopolamine-rich phenotypes, *D. metel* and a hybrid of *Duboisia* developed by Boehringer Ingelheim as an industrial source of scopolamine (personal communication), as well as a scopolamine-poor plant, *H. muticus*. In order to overexpress the tobacco *pmt* gene, we developed a binary system, consisting of a disarmed *Agrobacterium tumefaciens* C58C1, which contained the plasmid pRiA4 together with the plasmid pBMI. The latter plasmid carried the *pmt* gene, under the control of the promoter CaMV-35S, and the *npt*II gene. The results of the agroinfection showed that the *Agrobacterium* used in the three plant species had a high virulence. In all cases we obtained root lines with a high capacity for growth, similar to the root lines obtained after infection with a wild strain of *A. rhizogenes*, but the metabolic behavior changed considerably compared with the plant species. In the *Duboisia* hybrid the levels of *N*-methylputrescine (the direct product of the reaction catalyzed by PMT) of the engineered roots increased up to four-fold compared with wild type hairy roots, but the tropane alkaloids hyoscyamine and scopolamine did not increase significantly [73]. 

The results obtained from engineered roots of *H. muticus* and *D. metel* were also very different. As Moyano *et al.* [74] reports, in both species the overexpression of the *pmt* gene from tobacco under the control of the CaMV-35S promoter increased the hyoscyamine content, but while the production of scopolamine improved significantly in *Datura*, in *Hyoscyamus* the tropane alkaloid level remained similar to that of wild type hairy roots. In the engineered roots of *Duboisia*, alkaloid production was closely related to the presence of the 35S-*pmt* transgene, showing that ectopic expression of tobacco *pmt* increased the biosynthetic flux towards the tropane alkaloids and, consequently, the tropane alkaloid contents of transformed roots were significantly enhanced. In *A. belladonna*, it has been shown that regulation of the plant's endogenous *pmt* is pericycle specific [28]. It is evident that the transcriptional control by the 35S promoter of the transgenic *pmt* gene in the *Datura* root lines is not cell type specific. Furthermore, it may also indicate that the transgene allows the bypassing of the endogenous control of the metabolic flux to the alkaloids, which would take place as the first committed enzymatic step in their biosynthesis. Similar results have been obtained in *H. muticus* roots by Biondi *et al.* [75]. On the contrary, as already mentioned, in *Duboisia* and *A. belladonna* overexpression of the *pmt* gene only increased the accumulation of the direct metabolite *N*-methylputrescine [32,73], while the effect on the alkaloid level was marginal. These results, unlike those obtained by Moyano *et al.* [74] for *H. muticus* and *D. metel*, show that the operative regulatory pathways in tropane alkaloid plant species vary.

More recently, the tobacco *pmt* gene has been overexpressed in scopolamine-producing *H. niger* [76]. The engineered lines showed a significant increase in PMT activity and the contents of *N*-methylputrescine also increased more than five-fold, although the level of tropane alkaloids remained constant. It has also been demonstrated that the exposure of the roots to the elicitor methyl jasmonate increases the levels of polyamines as well as tropane alkaloids, and suggests that the C-flux for tropane alkaloid production could be restricted in later steps of the biosynthetic pathway.

## Overexpression of tropinone reductases

Another branching point in scopolamine biosynthesis is at the tropinone reductase level (see Figure 2). *Atropa*, *Datura*, *Hyoscyamus* and *Duboisia* contain additional tropane alkaloids, calystegines, which are characterized by the loss of a methyl group on the nitrogen bridge and by the presence of three to five hydroxyl groups on the tropane heterocycle [17]. Two separate tropinone reductases have been isolated and characterized from root cultures of several species, *D. stramonium* [77,78,79], *Hyoscyamus niger* [29,80] and *A. belladonna* [81] and the corresponding cDNAs were cloned [83,84]. The activity of the tropine-forming enzyme TRI (EC 1.1.1.206) could be flux-limiting for the biosynthesis of hyoscyamine and scopolamine, whereas TRII (EC 1.1.1.236) catalyzes the formation of pseudotropine and consequently drives the C-metabolic flux from tropane alkaloids to calystegines [84]. 

Overexpression of both tropinone reductases TRI and TRII have been achieved in *A. belladonna* hairy roots [17]. Engineered root lines with strong overexpression of the *trI* or *trII* gene from *D. stramonium* under the control of the CaMV 35S-promotor showed more enzyme activity of the respective reductase and a higher level of the enzyme products, tropine and pseudotropine. Strong expression of the *trI* gene was accompanied by a significant enhancement of the contents of hyoscyamine and scopolamine. On the contrary, calystegine levels were lower than in the control roots. These results show the effectiveness of the system for increasing scopolamine production in hairy roots and suggest that the tropane alkaloid and calystegine pathways compete for the C-flux. On the contrary, overexpression of the pseudotropine-forming reductase increased the accumulation of calystegines while the hyoscyamine content of the engineered roots remained constant [17].

## Overexpression of the *h6h* gene to increase the epoxidation of hyoscyamine into scopolamine

As mentioned above, the last step in the scopolamine biosynthetic pathway is the epoxidation of hyoscyamine catalyzed by hyoscyamine 6-β-hydroxylase (Figure 2). H6H, therefore, is a promising target enzyme that, if overexpressed in hyoscyamine-accumulating tissues, would result in increased scopolamine levels in transgenic plants or roots. In this way, several unattractive hyoscyamine-rich but scopolamine-poor plants, such as *Hyoscyamus* spp. could be converted into an industrial source of scopolamine [71].

The first example of how pharmaceutically important plants can be successfully altered by metabolic engineering was provided by Yun *et al.* [85]. Their research focused on *Atropa belladonna*, which is a hyoscyamine-rich phenotype. The *h6h* gene from *H. niger* was overexpressed in the target plant and the alkaloid pattern in a single primary transformed plant and its progenies showed elevated scopolamine contents, resulting in near to complete conversion of hyoscyamine to scopolamine in the mature plants. Working with hairy roots, Hashimoto *et al.* [86] showed that overexpression of the *h6h* transgene led to a higher production of scopolamine than hyoscyamine, the reverse of alkaloid production in wild-type roots. Similar results were achieved with *H. muticus* hairy roots overexpressing the same gene, in which the best transgenic clone had a 100-fold increase of scopolamine, while the hyoscyamine content remained unaltered [7]. These results indicate that overexpressing the *h6h* gene can be a way of increasing scopolamine production in hairy root cultures of those species that produce low levels of this alkaloid. However, the yields of scopolamine obtained so far are still too low for commercialization.

The previous experiments were mainly carried out with phenotypes that were poor in scopolamine and accumulated hyoscyamine as the principal alkaloid. In the case of a scopolamine-rich phenotype, such as the *Duboisia* hybrid (*D. myoporoides* x *D. leichhardtii*), overexpression of the *h6h* gene from *H. niger* under the control of the CaMV 35-S promoter significantly increased the scopolamine contents of the engineered root lines at the end of the culture period, with notable differences in alkaloid production observed among the different root lines established. The total alkaloid content (measured as hyoscyamine + scopolamine) was also higher in the root lines overexpressing the *h6h* gene compared with the typical hairy roots [87]. The results thus showed that overexpression of the 35S-*h6h* gene not only enhanced the capacity of root lines to convert hyoscyamine to scopolamine but also the total alkaloid production of these roots. 

In all the cases commented above, significant quantities of hyoscyamine remain in the root tissues without biotransformation into scopolamine. In contrast, engineered hairy root cultures of another scopolamine-poor genotype, *Atropa baetica*, overexpressing the 35S-*h6h* transgene, showed an altered alkaloid profile in which hyoscyamine, the main alkaloid in the plant, was entirely converted into scopolamine. In the best h6h clone, scopolamine accumulation increased 9-fold compared to plants, some of which was released into the liquid medium. Only negligible amounts of hyoscyamine were detected [88]. 

Recently, Zhang *et al.* [89] reported the simultaneous introduction and overexpression of genes encoding the rate-limiting upstream enzyme PMT and the downstream enzyme H6H of scopolamine biosynthesis in hairy root cultures of *H. niger*. Transgenic root lines expressing both *pmt* and *h6h* genes produced significantly higher levels of scopolamine compared with the wild-type roots. The best line produced 411 mg/L scopolamine, the highest scopolamine content achieved so far by a genetically engineered plant. This study on simultaneously engineering *pmt* and *h6h* genes in scopolamine-producing plant species has resulted in a significant enhancement of scopolamine accumulation in cultured hairy root lines. Overexpression of multiple biosynthetic genes or transcription factors that control the expression of genes in pathways targeted by bioengineering is a promising strategy to alter the accumulation of certain secondary metabolites.

## Biotransformation of hyoscyamine into scopolamine in transgenic tobacco hairy roots

Metabolic engineering can also provide techniques to transfer a whole metabolic pathway, or some of its steps, from one plant species to another. Rocha et al. [90] have simultaneously introduced two genes involved in tropane-alkaloid biosynthesis, *TR I* and *H6H*, in *Nicotiana tabacum*, expressed under the control of the CaMV 35S promoter. Detached leaves from the transgenic plants were fed with hyoscyamine and the expected H6H reaction product was generated. In addition, in most cases leaves of the transgenic plants showed higher nicotine contents than control plants, suggesting changes in the activity of the enzymes in the nicotine biosynthetic pathway.

We recently obtained three tobacco hairy root lines carrying the 35S-*h6h* gene from *Hyoscyamus niger*. The transformation was performed using a binary vector system based on *Agrobacterium rhizogenes*, as previously described by Palazón *et al*., [87], and the production of scopolamine in hairy roots was clearly correlated with the 35S-*h6h* transcript expression. The engineered *Nicotiana tabacum* hairy roots were studied for bioconversion after feeding the cultures with exogenous hyoscyamine. Engineered roots carrying the 35S-*h6h* transgene showed an efficient uptake of hyoscyamine (average of 95%) from the culture medium and also a higher rate of bioconversion of hyoscyamine to scopolamine (10-45%). Another important trait of this bioprocess was the remarkably high secretion of scopolamine from the roots, with up to 85% of the total scopolamine being released to the culture medium [91]. This contrasted with the normal metabolic behavior of tropane alkaloid-producing hairy roots in which the scopolamine remains accumulated in the root tissues [53]. 

**Figure 7:**
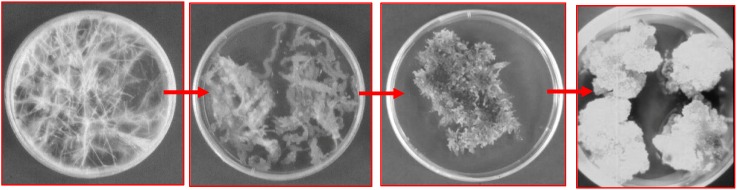
Dedifferentiation of tobacco hairy roots by subculturing in hormone-supplemented MS medium.

As mentioned previously, one of the most important obstacles for the industrial production of pharmaceuticals in biotechnological systems based on hairy root cultures is the scale-up to bioreactors. With the aim of scaling-up the biotransformation of hyoscyamine into scopolamine we obtained cell cultures derived from hairy roots overexpressing the *h6h* gene from *H. niger*. The hairy root cultures, obtained as described by Häkkinen *et al.* [91], were treated with indolacetic acid (11.5 μM) and kinetin (1 μM) to dedifferentiate the root tissues and obtain friable calli (Figure 7). After 3-5 subcultures in hormone-supplemented MS media the friable calli were transferred to the same liquid medium in order to obtain a fine cell suspension. The cell cultures were fed with hyoscyamine and 4 weeks later the amount of scopolamine produced was quantified by HPLC. The transgenic cell suspension cultures, like the hairy roots they derived from, showed a considerable capacity for the bioconversion of hyoscyamine into scopolamine (16%), and released it to the culture medium [92]. Although the scale-up from shake-flask to bioreactor culture usually results in reduced productivities, the transgenic cells grown in a 5-L turbine stirred tank reactor in a batch mode significantly increased the scopolamine accumulation. The total content (cell-associated+extracellular) of scopolamine was 35.5 mg/L, which was 1.6 times higher than that obtained in small-scale cultures. In this case almost 18% of the hyoscyamine added to the medium was transformed into scopolamine, which represented an increase of 65% with respect to the same alkaloid obtained by bioconversion in shake flasks.

## Future Challenges

Metabolic engineering, either alone or in combination with traditional cultivation techniques, provides the means to develop novel sources of plants with quantitatively and qualitatively improved pharmacological properties. However, the regulation towards the desired medicinal products requires a complete knowledge of the steps of the biosynthetic pathway and the respective cloned genes. Another problem is the difficulty of predicting the results of overexpressing a single or reduced number of genes, due to the frequent presence of multiple rate-limiting steps. Unfortunately, as expressed by the title of Humphrey and O’Hagan’s paper [16], “Tropane alkaloid biosynthesis. A century old problem unresolved”, several aspects of scopolamine biosynthesis remain unknown and we are also far from understanding the mechanism of their regulation. It is therefore necessary to consider not only the genes coding for enzymes involved in individual steps of the pathway, but also the homeotic genes controlling the transcription of numerous genes involved in the regulation of the whole pathway and interconnecting cellular pathways [11] 

Oksman-Caldentey and Inzé [93] have described a novel gene discovery platform based on functional genomics. When comparing transcriptomic and metabolomic profiling, it is possible to obtain a large number of genes whose expression correlates with the accumulation of secondary metabolites. In this context, the elicitation of tobacco cell cultures with methyl jasmonate has permitted the identification of more than 600 tags modulated by the elicitor and probably related with the tobacco alkaloid pathway [94]. Similar studies carried out with plant cell cultures of *Catharanthus roseus*, a plant species that produces more than 120 alkaloids, have established a correlation within the transcriptomic profile of 417 tags and the metabolic accumulation of 178 alkaloids [95]. The genomic approach applied to scopolamine biosynthesis could constitute a potent tool to elucidate this unresolved pathway in its entirety with the aim of increasing the biotechnological production of this alkaloid. The transfer of part of a metabolic pathway from one plant species to another could also be an excellent system to obtain novel phytochemical compounds with new or improved biological activities.
